# Diagnostic value of ^18^F-FDG PET-CT in detecting malignant peripheral nerve sheath tumors among adult and pediatric neurofibromatosis type 1 patients

**DOI:** 10.1007/s11060-021-03936-y

**Published:** 2022-01-13

**Authors:** Ritch T. J. Geitenbeek, Enrico Martin, Laura H. Graven, Martijn P. G. Broen, Monique H. M. E. Anten, Jochem A. J. van der Pol, Cornelis Verhoef, Walter Taal

**Affiliations:** 1 Department of Plastic and Reconstructive Surgery G04.126, University Medical Center Utrecht, PO Box 85060, 3508 AB Utrecht, The Netherlands; 2 Department of Surgical Oncology, Erasmus Medical Center Cancer Institute, Rotterdam, Netherlands; 3 Department of Radiology and Nuclear Medicine, Erasmus Medical Center Cancer Institute, Rotterdam, Netherlands; 4 Department of Neurology, Maastricht University Medical Center, Maastricht, Netherlands; 5 Department of Radiology and Nuclear Medicine, Maastricht University Medical Center, Maastricht, Netherlands; 6 Department of Neurology, Erasmus Medical Center Cancer Institute, Rotterdam, Netherlands

**Keywords:** ^18^F-FDG PET-CT, Neurofibroma, Neurofibromatosis, MPNST, Diagnostic algorithm

## Abstract

**Purpose:**

Detecting malignant peripheral nerve sheath tumors (MPNSTs) remains difficult. ^18^F-FDG PET-CT has been shown helpful, but ideal threshold values of semi-quantitative markers remain unclear, partially because of variation among scanners. Using EU-certified scanners diagnostic accuracy of ideal and commonly used ^18^F-FDG PET-CT thresholds were investigated and differences between adult and pediatric lesions were evaluated.

**Methods:**

A retrospective cohort study was performed including patients from two hospitals with a clinical or radiological suspicion of MPNST between 2013 and 2019. Several markers were studied for ideal threshold values and differences among adults and children. A diagnostic algorithm was subsequently developed.

**Results:**

Sixty patients were included (10 MPNSTs). Ideal threshold values were 5.8 for SUVmax (sensitivity 0.70, specificity 0.92), 5.0 for SUVpeak (sensitivity 0.70, specificity 0.97), 1.7 for TLmax (sensitivity 0.90, specificity 0.86), and 2.3 for TLmean (sensitivity 0.90, specificity 0.79). The standard TLmean threshold value of 2.0 yielded a sensitivity of 0.90 and specificity of 0.74, while the standard SUVmax threshold value of 3.5 yielded a sensitivity of 0.80 and specificity of 0.63. SUVmax and adjusted SUV for lean body mass (SUL) were lower in children, but tumor-to-liver ratios were similar in adult and pediatric lesions. Using TLmean > 2.0 or TLmean < 2.0 and SUVmax > 3.5, a sensitivity and specificity of 1.00 and 0.63 can be achieved.

**Conclusion:**

^18^F-FDG PET-CT offers adequate accuracy to detect MPNSTs. SUV values in pediatric MPNSTs may be lower, but tumor-to-liver ratios are not. By combining TLmean and SUVmax values, a 100% sensitivity can be achieved with acceptable specificity.

**Supplementary Information:**

The online version contains supplementary material available at 10.1007/s11060-021-03936-y.

## Introduction

Peripheral nerve sheath tumors (PNSTs) are relatively common and include both benign and malignant tumors. Schwannomas are the most common benign nerve sheath tumors (BPNSTs) and neurofibromas make up the largest proportion of remaining BPNSTs [1, 2]. Nerve sheath tumors may arise sporadically or in association with neurofibromatosis. Neurofibromatosis type 1 (NF1) patients are at increased risk for developing PNSTs, with often high body tumor burden of neurofibromas [1–4]. Importantly, these neurofibromas may act as precursor lesions and can transform into malignant peripheral nerve sheath tumors (MPNSTs) [5]. MPNSTs are aggressive soft tissue sarcomas (STS), accounting for 2–3% of all STS [6, 7]. Although MPNSTs are rare in the common population, NF1 patients have an 8–13% lifetime risk of developing an MPNST. MPNSTs generally have poor clinical outcomes, being the leading cause of mortality in NF1 patients [8, 9]. The median survival of localized disease ranges from 5–6 years, demanding aggressive treatment [10, 11]. Surgical resection is the only curative therapeutic option improving survival as MPNSTs respond poorly to chemo- and radiotherapy [10–12]. While the resection of MPNSTs commonly results in high postoperative morbidity and motor deficits, BPNSTs may be removed by intracapsular resections, minimizing neurologic damage [13–15]. BPNSTs only require resection in selected cases, making adequate preoperative differentiation crucial.

^18^F-FDG PET-CT, using standardized uptake values (SUVs) and tumor-to-liver ratios as semi-quantitative metabolic imaging markers, has been increasingly used as a non-invasive diagnostic tool for the characterization of PNSTs in NF1 patients. However, ideal parameters and their corresponding thresholds have yet to be elucidated [16]. There is large variation in current literature regarding this matter, part of which might be caused by variation among scanners and scanning protocols [17–20]. Suggested optimal threshold values of semi-quantitative parameters vary greatly, but the SUVmax threshold of ≥ 3.5 is commonly cited [21–24]. However, its value has been doubted since it may provide high false positive rates [22]. Additional concerns rise among scanning in pediatric NF1 populations, as few studies have investigated the diagnostic accuracy in this subpopulation. By using European Association of Nuclear Medicine (EANM) Research Ltd. (EARL) protocol certified scanners, results are reproducible for any center utilizing a scanner of that kind.

Given current uncertainties of accurately distinguishing MPNSTs and BPNSTs using ^18^F-FDG PET-CT, this study investigated the diagnostic accuracy of optimal and commonly used thresholds of semi-quantitative ^18^F-FDG PET-CT markers using EARL certified scanners and evaluated possible differences between adult and pediatric populations.

## Methods

### Study population

Patient data was retrospectively collected from two neurofibromatosis expertise centers. Patients with NF1 (fulfilling the NIH criteria and/or genetically proven) who underwent ^18^F-FDG PET-CT examination for suspected MPNST based on clinical symptoms and/or radiological examination were included. The EARL protocol is used for performance harmonization for semi-quantitative imaging markers of ^18^F-FDG PET-CT, enabling comparison of imaging markers among patients and sites, regardless of the ^18^F-FDG PET-CT used. To increase homogeneity between imaging only patients following EARL protocol were included, thus only patients that underwent scans after 2013 were included. Patients with BPNSTs, either suspected or concluded by biopsy, with less than 12 months follow-up were excluded. Patients receiving treatment consisting of radiotherapy, chemotherapy or surgical excision of the lesion prior to ^18^F-FDG PET-CT were excluded as this may alter tumor imaging features. Patient data was obtained from electronic medical files including demographical information, histopathological outcomes, and (semi-quantitative) scan characteristics. This study was approved by the Ethics Committee of both participating centers with waiver of individual patient consent.

### Image acquisition

**Fig. 1:**
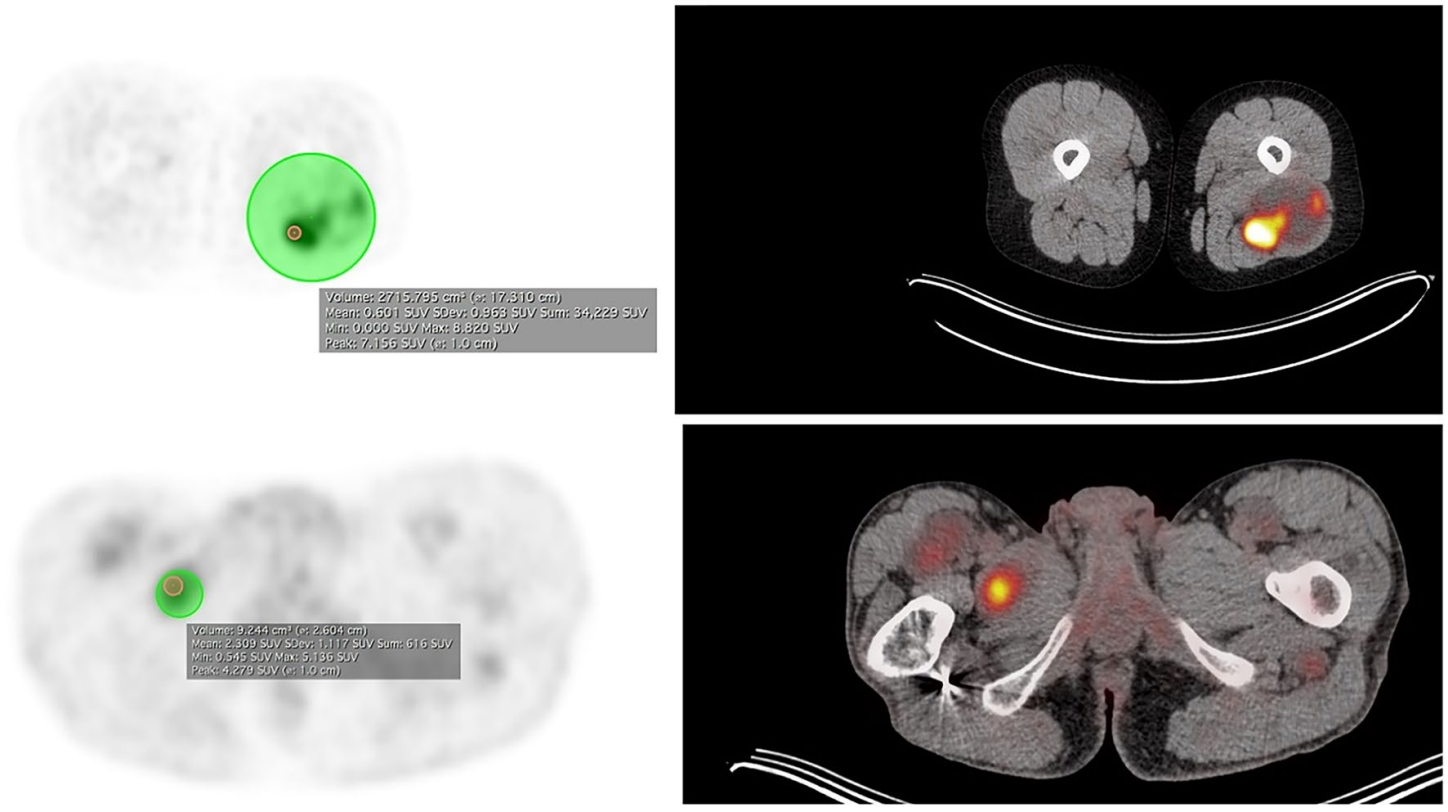
Maximum, mean, and peak standardized uptake values determined by drawing a volume of interest around the target lesion. Imaging displaying maximum, mean, and peak standardized uptake values determined by drawing a volume of interest around the target lesion in MPNST and neurofibroma. *MPNST* malignant peripheral nerve sheath tumor; *SUV* standard uptake value

^18^F-FDG PET-CT scans were performed using a Siemens Biograph mCT PET/CT scanner (Siemens Healthineers, Erlangen, Germany) and Philips Gemini 64 TOF (Philips Medical Systems International BV, Best, The Netherlands). After fasting for approximately 4–6 h the patients received intravenous administration of ^18^F-2-fluoro-2-deoxy-d-glucose (FDG). In adults the dose of FDG in MBq was based on weight in one center and on weight adjusted to surface body area (ranging 113–385) in the other. Pediatric patients received weight-dependent administration of FDG based on the pediatric dose card of the EANM [25]. Administration of tracer took place after confirming blood glucose levels were within normal range. If blood glucose levels were greater than 10 mmol/L, the study was rescheduled. Whole body attenuation corrected images were acquired approximately 60 min after tracer injection. During this uptake phase, patients were instructed to rest in a warm, dimly lit room with minimal stimulation. According to scanning protocol, first a whole-body low dose CT was acquired for attenuation correction and localization purposes (120 kV, Quality reference mAs 40, rotation time 0.5 s, pitch of 0.8 mm, slice thickness of 3 mm; reconstructed slice thickness 3 mm). Directly after the low dose CT, PET acquisition started in list-mode, using 6 to 7 bed positions per patient (from skull base to inguinal region). All scans were corrected for scatter and attenuation using the low dose CT and reconstructed using ordered subset expectation maximization (OSEM) and Time of Flight (TOF). Logistic time constraints warranted delayed imaging was performed after 3 h. In the neurofibromatosis expertise centers, semi-quantitative analysis was performed by a nuclear medicine physician with over 3 years of experience, blinded to both clinical history and pathology results. Maximum, mean, and peak standardized uptake values (SUVmax, SUVmean, and SUVpeak) were determined by drawing a volume of interest (VOI) around the target lesion or in the liver as reference (Fig. 1). Tumor-to-liver ratios were determined by drawing a VOI with a diameter of 3 cm in the center of the right liver lobe. Care was taken that the whole VOI was inside the liver. The SUVmax and mean of this region were measured.

### Histological analysis

Histology was considered gold standard and was performed according to institutional standards. Tumors were classified as typical neurofibroma, atypical neurofibroma, or malignant peripheral nerve sheath tumor, using established pathologic criteria [20, 26, 27].

### Statistical analysis

The following semi-quantitative imaging markers were analyzed for potential use to differentiate malignant transformation in neurofibromas: SUVmax, SUVpeak, SUVmax adjusted to lean body mass (SULmax), SUVpeak adjusted to lean body mass (SULpeak), delayed SUVmax, delayed SUVpeak, delayed SULmax, delayed SULpeak, TLmax, and TLmean. Lean body mass (LBM) was calculated using Janmahasatian’s formula [25–29]. Receiver operating characteristic (ROC) analysis was performed for each semi-quantitative imaging marker and optimal threshold values were determined using Youden’s index. Ideal threshold sensitivity, specificity, positive likelihood ratio (pLR), and negative likelihood ratio (nLR) were determined. Diagnostic accuracy was described using area under the receiver operating curve (AUC). Optimization of the diagnostic algorithm was performed using commonly used imaging markers SUVmax and TLmean. Performance of commonly used threshold values for SUVmax (3.0–6.0) and TLmean (1.5–3.0) was assessed. Steps of 0.5 were used to improve generalizability. Additionally, threshold values yielding 100% sensitivity or 100% specificity were assessed. Combinations of these parameters were manually assessed to identify the diagnostic algorithm with highest sensitivity and acceptable specificity. Patients were stratified by age (adults vs. children) and subgroup analysis was performed for MPNSTs and BPNSTs. Nine patients received more than one ^18^F-FDG PET-CT. Differences in PNSTs and between subgroups were analyzed using chi-square test for categorical variables and for continuous variables a one-way test/t-test depending on normality of distribution based on the Shapiro–Wilk test. Additionally, Kruskal–Wallis or Wilcoxon test were used, depending on distribution. As recent literature indicates that PNSTs are at risk of undergoing malignant transformation at any point in time, each tumor was investigated for malignant transformation at every ^18^F-FDG PET-CT independently of previous measurements [23, 30–32]. Typical and atypical neurofibromas were evaluated together as they are both considered benign lesions. Statistical significance was established for p-values < 0.05. All statistical analyses were performed using R version 4.0.3 (R Core Team 2020).

## Results

### Study population

Sixty patients were included, undergoing ^18^F-FDG PET-CT examinations for seventy tumors, 10 MPNSTs and 60 BPNSTs (Table 1). Forty lesions were found in females and thirty in males. Nineteen of seventy lesions had delayed scans, of which 3 MPNSTs. Fifteen lesions were evaluated in children (≤ 18 years). Mean duration of follow-up was 3.5 ± 1.6 years. At last follow-up, 7 MPNST patients and 4 BPNST patients were deceased.

**Table 1:** Patient characteristics of study population

	MPNSTs	BPNSTs
Total lesions (%)	10 (14.3)	60 (85.7)
Gender		
Female	7 (70.0)	33 (55.0)
Male	3 (30.0)	27 (45.0)
Age		
≤ 18 years	2 (20.0)	13 (21.7)
> 18 years	8 (80.0)	47 (78.3)
Mean age	43 (± 18.5)	37 (± 15.4)
Diagnosis		
Resection	9 (90)	18 (30.0)
Biopsy	1 (10)	10 (16.7)
Follow-up > 12 months	–	32 (53.3)
Mean follow-up	1.5 (± 0.7)	3.5 (± 1.6)

Table displaying patient characteristics of included population. Mean age in years ± SD. Diagnosis, n (%) refers to number of lesions diagnosed based on biopsy or resection. Follow up > 12 months refers to lesions diagnosed as benign based on being clinically silent after a minimum of 12 months of clinical and radiological follow-up. Mean length of follow-up in years ± SD. *BPNST* benign peripheral nerve sheath tumor; *MPNST* malignant peripheral nerve sheath tumor

### Optimal threshold values

The optimal threshold for SUVmax was ≥ 5.82 (AUC = 0.88) (Table 2, Supplementary Fig. 1). Sensitivity and specificity were 0.70 and 0.92, respectively. pLR and nLR were 8.26 and 0.33, respectively. Optimal threshold for SULmax was ≥ 8.83 (AUC = 0.86). Sensitivity and specificity were 0.67 and 0.90, respectively. pLR and nLR were 6.56 and 0.37, respectively. Optimum threshold for TLmean was ≥ 2.31 (AUC = 0.91). Sensitivity and specificity were 0.90 and 0.79, respectively. pLR and nLR were 4.35 and 0.13, respectively. Optimum threshold for delayed SUVmax was ≥ 2.53 (AUC = 0.81, Supplementary Fig. 2). Sensitivity and specificity were 1.00 and 0.56, respectively. pLR and nLR were 2.29 and 0.00, respectively. Optimum threshold for delayed SULmax was ≥ 3.42 (AUC = 0.69). Sensitivity and specificity were 1.00 and 0.50, respectively. pLR and nLR were 2.00 and 0.00, respectively.

**Table 2:** ROC analysis of diagnostic accuracy of semi-quantitative imaging markers

	Threshold value*	Sens	Spec	pLR	nLR	AUC
SUVmax	5.82	0.70	0.92	8.26	0.33	0.88 (0.75, 1.00)
SUVpeak	5.00	0.70	0.97	20.65	0.31	0.88 (0.75, 1.00)
SULmax	8.83	0.67	0.90	6.56	0.37	0.86 (0.72, 0.99)
SULpeak	7.58	0.67	0.98	39.33	0.34	0.86 (0.72, 1.00)
Delayed SUVmax	2.53	1.00	0.56	2.29	0.00	0.81 (0.53, 1.09)
Delayed SUVpeak	2.08	1.00	0.63	2.67	0.00	0.85 (0.61, 1.10)
Delayed SULmax	3.42	1.00	0.50	2.00	0.00	0.69 (0.28, 1.10)
Delayed SULpeak	2.81	1.00	0.63	2.67	0.00	0.78 (0.44, 1.12)
TLmax	1.66	0.90	0.86	6.53	0.12	0.92 (0.84, 1.01)
TLmean	2.31	0.90	0.79	4.35	0.13	0.91 (0.81, 1.00)

Table showing ROC analysis of diagnostic accuracy of semi-quantitative imaging markers. *Optimal threshold obtained through Youden’s method. *AUC* area under the receiver operating curve; *nLR* negative likelihood ratio; *pLR* positive likelihood ratio; *sens* sensitivity; spec specificity; *SUV* standard uptake value; *SUL* standard uptake value adjusted for lean body mass; *TLmax* tumor-to-liver maximal ratio; *TLmean* tumor-to-liver mean ratio

### Differences between adults and children

Statistically significant differences between adults and children were found in MPNSTs for mean SUVmax (11.56 vs. 3.10, p = 0.037) and SUVpeak (7.48 vs. 2.14, p = 0.037), but not in BPNSTs (Table 3). By adjusting for LBM, uptake values for pediatric MPNSTs were still significantly lower: SULmax (15.53 vs. 4.25, p = 0.040) and SULpeak (10.59 vs. 2.92, p = 0.040). Proportional values (TLmax and TLmean) were not statistically lower in pediatric MPNSTs.

**Table 3:** Semi-quantitative imaging markers stratified by age

	Adult	Child	p-value
SUVmax			
MPNST	11.56 [8.07–12.53]	3.10 [2.96–3.25]	0.037
BPNST	2.83 [1.56–4.41]	2.22 [1.95–3.12]	0.609
Combined	3.56 [1.77–4.65]	2.24 [2.00–3.26]	0.233
SUVpeak			
MPNST	7.48 [6.62–10.49]	2.14 [2.11–2.16]	0.037
BPNST	2.10 [1.14–3.56]	1.61 [1.36–2.09]	0.464
Combined	2.68 [1.20–3.84]	1.72 [1.37–2.14]	0.163
Delayed SUVmax			
MPNST	9.59 [8.30–10.89]	2.53 [2.53–2.53]	0.221
BPNST	2.85 [1.38–4.52]	2.15 [1.53–2.41]	0.777
Combined	3.60 [1.46–7.00]	2.28 [1.69–2.50]	0.430
Delayed SUVpeak			
MPNST	7.44 [6.29–8.59]	2.08 [2.08–2.08]	0.221
BPNST	1.85 [1.08–3.33]	1.65 [1.24–1.68]	0.777
Combined	2.57 [1.12–4.58]	1.67 [1.34–1.98]	0.483
TLmax			
MPNST	3.35 [2.74–5.40]	1.76 [1.71–1.81]	0.192
BPNST	0.95 [0.56–1.43]	0.80 [0.70–1.09]	0.675
Combined	1.09 [0.60–1.59]	0.96 [0.72–1.56]	0.947
TLmean			
MPNST	5.97 [3.95–7.57]	2.33 [2.32–2.35]	0.117
BPNST	1.38 [0.82–2.15]	1.13 [0.98–1.56]	0.904
Combined	1.62 [0.87–2.70]	1.21 [1.01–2.16]	0.750
SULmax			
MPNST	15.53 [11.65–18.51]	4.25 [4.02–4.48]	0.040
BPNST	3.93 [2.47–6.38]	2.73 [2.37–4.44]	0.265
Combined	4.72 [2.60–6.82]	3.29 [2.48–4.57]	0.105
SULpeak			
MPNST	10.59 [8.23–15.54]	2.92 [2.87–2.98]	0.040
BPNST	3.45 [1.64–5.06]	2.12 [1.65–2.90]	0.235
Combined	4.09 [1.78–5.58]	2.54 [1.68–2.97]	0.082
Delayed SULmax			
MPNST	10.62 [10.62–10.62]	3.42 [3.42–3.42]	0.317
BPNST	3.86 [1.91–6.69]	2.39 [1.93–3.91]	0.610
Combined	4.94 [1.95–7.69]	2.90 [2.04–3.79]	0.454
Delayed SULpeak			
MPNST	7.80 [7.80–7.80]	2.81 [2.81–2.81]	0.317
BPNST	2.50 [1.50–5.28]	1.88 [1.55–2.68]	0.610
Combined	3.13 [1.55–6.03]	2.28 [1.63–2.78]	0.512

Table displaying semi-quantitative imaging markers stratified by age, with results for all PNSTs combined, for MPNST subgroup, and BPNST subgroup. Normal distributed data were described using means and standard deviations. Non-normal distributed data were described using medians and interquartile ranges. *BPNST* benign peripheral nerve sheath tumor; *MPNST* malignant peripheral nerve sheath tumor; *SUV* standard uptake value; *SUL* standard uptake value adjusted for lean body mass; *TLmax* tumor-to-liver maximal ratio; *TLmean* tumor-to-liver mean ratio

### PET algorithm

**Fig. 2:**
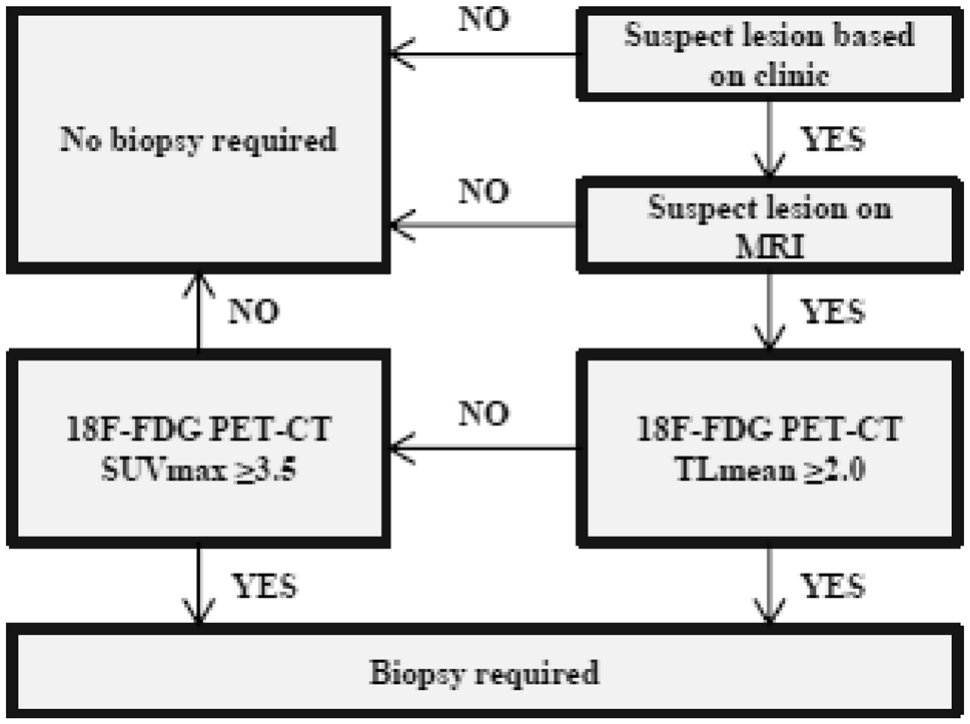
Diagnostic algorithm using TLmean and SUVmax. Diagnostic algorithm for optimal diagnostic work-up by performing biopsies in lesions with threshold of TLmean ≥ 2.0 or TLmean < 2.0 and SUVmax ≥ 3.5. *MRI* magnetic resonance imaging; *SUV* standard uptake value; *TL* tumor-to-liver ratio

An SUVmax of 2.8 yielded 100% sensitivity (Supplementary Tables 1 and 2). An SUVmax of 7.3 yielded 100% specificity. The commonly used threshold of ≥ 3.5 for SUVmax yielded 80% sensitivity with 63% specificity. A TLmean of 1.6 yielded 100% sensitivity. A TLmean of 4.8 yielded 100% specificity. A commonly used threshold of ≥ 2.0 for TLmean yielded 90% sensitivity with 74% specificity. As TLmean ≥ 2.0 offers higher accuracy than ≥ 3.5 SUVmax, and values do not differ significantly between adults and children, an optimal diagnostic work-up can be achieved by performing biopsies in lesions with threshold of TLmean ≥ 2.0 or TLmean < 2.0 and SUVmax ≥ 3.5 (Supplementary Table 3). This diagnostic algorithm resulted in 100% sensitivity and 63% specificity, requiring 22/60 BPNSTs to undergo biopsy (Fig. 2). Additionally, using the optimal threshold of TLmean found in this study (≥ 2.3), specificity may be increased to 65%, resulting in one less BPNST requiring biopsy.

## Discussion

This retrospective study found that PET scans offer adequate accuracy for detecting malignant transformation of neurofibromas both in adults and children. Combining SUVmax and TLmean threshold values in a diagnostic algorithm increases specificity while retaining 100% sensitivity.

### Optimal thresholds in PET scans

In the past decades, ^18^F-FDG PET-CT scans have increasingly been used to detect malignancy in NF1 patients. Though numerous studies aimed to identify ideal semi-quantitative imaging markers, ideal thresholds for detecting MPNSTs vary across studies. Differences in reported SUV measurements may occur due to different types of scanners and protocols being used. To diminish variations across scanners, criteria were formulated by the EARL to improve reproducibility of evaluated thresholds. Additionally, proportional SUV values as the TL ratio are proposed to reduce measurement variations. The most commonly evaluated characteristics for detection of malignant transformation of PNSTs are SUVmax and TLmean. Studies evaluating SUVmax reported ideal thresholds varying from 2.35 to 6.1 [20, 25–27, 31, 33–40]. Studies evaluating TL ratio reported ideal thresholds varying from 1.4 to 3.0 [17, 25, 31, 35, 37, 39, 41]. This study found ideal threshold values for SUVmax and TL ratio consistent with those reported in literature and delayed imaging did not improve diagnostic accuracy. However, using these thresholds some MPNSTs may be missed.

### Children vs. adult populations

Malignant transformation of neurofibromas also occurs in children [12, 42]. As detection of MPNST at early stages could increase the possibility of curative resections, frequent and serial imaging for surveillance of lesions is often performed. Conversely, this practice may possibly lead to harmful long term radiation effects [22, 35, 39, 43, 44]. Unfortunately, only few published ^18^F-FDG PET-CT studies have included children for analysis and no analysis has been performed comparing imaging marker values between adult and pediatric NF1 patients. Studies that combined data from both adults and children with NF1 found an optimal threshold value of SUVmax ranging from 3.90 to 4.00 with sensitivity ranging from 82 to 100% and specificity ranging from 66 to 94% [25–27]. Studies including only adult NF1 patients found a wider range of optimal threshold values for SUVmax ranging from 1.8 to 7.0, suggesting that children may have lower SUVmax values compared to adults [20–23, 28, 30–33, 35, 39, 41, 42, 44–46]. It is suggested that SUV values in adults may be higher, as the administered dose is adjusted by weight and since adults have comparably more fat tissue which has relatively low FDG, the uptake in lesions and normal organs is higher. Adjusting SUV to lean body mass may correct for body composition as a contributing factor for SUV differences found between adult and pediatric patients. Recent studies have investigated the use of SUL using James’s formula to improve diagnostic accuracy in differentiation of PNSTs in adult population [20, 29, 39, 42, 47, 48]. This study adjusted SUV to lean body mass using a recently proposed formula by Janmahasatian, as it is suggested to be more accurate for use in children [25–29]. Significantly lower SUVmax and SUVpeak values in MPNSTs in children were found. However, after adjusting for lean body mass uptake values of SUVmax and SUVpeak remained significantly lower in MPNSTs in children, suggesting it is less likely that differences in body composition significantly contribute to SUV differences found between adults and children [29]. Though based on only 2 MPNSTs, significantly lower SUV values were found in children. This may be due to the large spread in uptake values in adults, which require relatively low SUVmax thresholds. Nevertheless, based on the significant differences in SUV values between adults and children, caution should be taken in interpreting SUV thresholds on their own in children.

### Optimal PET algorithm

A threshold of 3.5 for SUVmax has often been proposed as the ideal threshold [21–23]. A recent meta-analysis pooled individual level patient data from 11 different study populations and found a threshold of 3.5 provided the highest sensitivity (0.99) and acceptable specificity (0.75) [24]. Arguments against using this threshold often consisted of the low specificity it offered. This study found a sensitivity of 0.80 and specificity of 0.63 using a threshold of 3.5 for SUVmax. In this study, TLmean yielded slightly better accuracy (0.92) compared to SUVmax, while there was no significant difference between adults and children in proportional values. Contrasting to previous studies, the current study combined the use of SUVmax and TLmean, proposing an algorithm aimed to achieve optimal sensitivity while retaining acceptable specificity. Using a threshold of TLmean ≥ 2.0 or TLmean < 2.0 and SUVmax ≥ 3.5, sensitivity of 1.00 was achieved and specificity of 0.63. As TL values did not differ between the adult and pediatric population, there does not seem to be a rationale to have separate diagnostic algorithms. Using single semi-quantitative imaging markers, sensitivity of 1.00 is often not achieved or comes at the cost of lower specificity. A single marker’s threshold may also be less reproducible in other populations.

### Strengths and limitations

This study is limited by its relatively small population, which is mainly a result of the strict inclusion criteria. The inclusion of symptomatic lesions and EARL adhering scans only, is stricter than previous studies. As EARL criteria were adapted in both participating centers only in 2013 and a follow-up of a year for benign lesions was required, the study period was relatively short. Therefore, subgroup analysis of pediatric patients should be interpreted with caution. Despite these limitations, the results of this study are reproducible for any center using PET-scanners that adhere to EARL criteria. Additionally, this study used a combination of SUVmax and TLmean and developed an optimal diagnostic work-up algorithm to identify all MPNSTs while minimalizing the number of false positives. To the best of our knowledge, this is the first study to compare semi-quantitative imaging marker values between adult and pediatric patients. This study found that while SUVmax and SUL were significantly lower for MPNSTs in children, TL values were not. Based on the findings of this study, future research should investigate several knowledge gaps. First, the semi-quantitative characteristics evaluated in this study should be validated in large prospective cohort studies with PET scanners adhering to EARL criteria. This may identify ideal threshold values for accurate detection of malignant transformation of PNSTs. Secondly, the use of the proposed diagnostic algorithm should be replicated in a large database of adult and pediatric NF1 patients. Additionally, SUV values of semi-quantitative imaging markers in adult and pediatric NF1 patients should be studied too. Though adjusting optimal threshold values based on age did not impact the diagnostic accuracy of the proposed algorithm, potential differences in diagnostic accuracy between these populations may necessitate different diagnostic guidelines nevertheless. Altogether, the results from these studies will provide a framework that may enable optimal diagnostic algorithms to be formulated. This study only assessed the diagnostic accuracy of ^18^F-FDG PET-CT. A recently published meta-analysis reported that although conventional MRI yields varying degrees of accuracy, some studies have shown high accuracies in functional MRI [24]. Though further research is required on this modality, reducing the need for ^18^F-FDG PET-CT may diminish radiation exposure that accumulates due to numerous follow-up scans necessary in NF1 patients prone to tumorigenesis.

## Conclusion

In EARL adhering PET-scanners, semi-quantitative imaging markers offer acceptable diagnostic accuracy for detecting malignant transformation of PNSTs in NF1. An algorithm was proposed, combining SUVmax and TLmean, which maximizes sensitivity while simultaneously reducing the number of false positives, thus reducing the number of unnecessary biopsies. This algorithm can readily be used in any center using EARL adhering PET-scanners. In pediatric MPNSTs SUVmax values were significantly lower even after correction for lean body mass, yet TL values were similar to adult cases. These potential differences between uptake values of adults and children did not impact the diagnostic algorithm.

## Supplementary Information

Below is the link to the electronic supplementary material.

Supplementary file1 (DOCX 68 kb)

Supplementary file2 (DOCX 25 kb)

## Data Availability

The datasets generated during and/or analysed during the current study are available from the corresponding author on reasonable request.

## References

[1] Kim DH, Murovic JA, Tiel RL, Moes G, Kline DG (2005). A series of 397 peripheral neural sheath tumors: 30-year experience at Louisiana State University Health Sciences Center. J Neurosurg.

[2] Montano N, D’Alessandris QG, D’Ercole M (2016). Tumors of the peripheral nervous system: analysis of prognostic factors in a series with long-term follow-up and review of the literature. J Neurosurg.

[3] Friedman JM (1999). Epidemiology of neurofibromatosis type 1. Am J Med Genet.

[4] Listernick R, Charrow J (1990). Neurofibromatosis type 1 in childhood. J Pediatr.

[5] Staedtke V, Bai R-Y, Blakeley JO (2017). Cancer of the peripheral nerve in neurofibromatosis type 1. Neurotherapeutics.

[6] Le Guellec S, Decouvelaere A-V, Filleron T (2016). Malignant peripheral nerve sheath tumor is a challenging diagnosis: a systematic pathology review, immunohistochemistry, and molecular analysis in 160 patients from the french sarcoma group database. Am J Surg Pathol.

[7] Brennan MF, Antonescu CR, Moraco N, Singer S (2014). Lessons learned from the study of 10,000 patients with soft tissue sarcoma. Ann Surg.

[8] Evans DGR, Baser ME, McGaughran J, Sharif S, Howard E, Moran A (2002). Malignant peripheral nerve sheath tumours in neurofibromatosis 1. J Med Genet.

[9] Ferner RE, Huson SM, Thomas N (2007). Guidelines for the diagnosis and management of individuals with neurofibromatosis. J Med Genet.

[10] Valentin T, Le Cesne A, Ray-Coquard I (2016). Management and prognosis of malignant peripheral nerve sheath tumors: the experience of the French Sarcoma Group (GSF-GETO). Eur J Cancer.

[11] Martin E, Coert JH, Flucke UE (2019). A nationwide cohort study on treatment and survival in patients with malignant peripheral nerve sheath tumours. Eur J Cancer.

[12] Stucky C-CH, Johnson KN, Gray RJ (2012). Malignant peripheral nerve sheath tumors (MPNST): the mayo clinic experience. Ann Surg Oncol.

[13] Nelson CN, Dombi E, Rosenblum JS (2020). Safe marginal resection of atypical neurofibromas in neurofibromatosis type 1. J Neurosurg.

[14] Hajiabadi MM, Campos B, Sedlaczek O (2020). Interdisciplinary approach allows minimally invasive, nerve-sparing removal of retroperitoneal peripheral nerve sheath tumors. Langenbeck’s Arch Surg.

[15] Dunn GP, Spiliopoulos K, Plotkin SR (2013). Role of resection of malignant peripheral nerve sheath tumors in patients with neurofibromatosis type 1: clinical article. J Neurosurg.

[16] Tovmassian D, Abdul Razak M, London K (2016). The role of [18F]FDG-PET/CT in predicting malignant transformation of plexiform neurofibromas in neurofibromatosis-1. Int J Surg Oncol.

[17] Salamon J, Veldhoen S, Apostolova I (2014). F-18-FDG PET/CT for detection of malignant peripheral nerve sheath tumours in neurofibromatosis type 1: tumour-to-liver ratio is superior to an SUVmax cut-off. Eur Radiol.

[18] Salamon J, Derlin T, Bannas P (2013). Evaluation of intratumoural heterogeneity on 18F-FDG PET/CT for characterization of peripheral nerve sheath tumours in neurofibromatosis type 1. Eur J Nucl Med Mol Imaging.

[19] Salamon J, Papp L, Tóth Z (2015). Nerve sheath tumors in neurofibromatosis type 1: assessment of whole-body metabolic tumor burden using F-18-FDG PET/CT. PLoS ONE.

[20] Ahlawat S, Blakeley JO, Rodriguez FJ, Fayad LM (2019). Imaging biomarkers for malignant peripheral nerve sheath tumors in neurofibromatosis type 1. Neurology.

[21] Derlin T, Tornquist K, Münster S (2013). Comparative effectiveness of 18F-FDG PET/CT versus whole-body MRI for detection of malignant peripheral nerve sheath tumors in neurofibromatosis type 1. Clin Nucl Med.

[22] Warbey VS, Ferner RE, Dunn JT, Calonje E, O’Doherty MJ (2009). FDG PET/CT in the diagnosis of malignant peripheral nerve sheath tumours in neurofibromatosis type-1. Eur J Nucl Med Mol Imaging.

[23] Ferner RE, Golding JF, Smith M (2008). [18F]2-fluoro-2-deoxy-D-glucose positron emission tomography (FDG PET) as a diagnostic tool for neurofibromatosis 1 (NF1) associated malignant peripheral nerve sheath tumours (MPNSTs): a long-term clinical study. Ann Oncol.

[24] Martin E, Geitenbeek RTJ, Coert JH (2020). A Bayesian approach for diagnostic accuracy of malignant peripheral nerve sheath tumors: a systematic review and meta-analysis. Neuro Oncol.

[25] Azizi AA, Slavc I, Theisen BE (2018). Monitoring of plexiform neurofibroma in children and adolescents with neurofibromatosis type 1 by [(18) F]FDG-PET imaging. Is it of value in asymptomatic patients. Pediatr Blood Cancer.

[26] Moharir M, London K, Howman-Giles R (2010). Utility of positron emission tomography for tumour surveillance in children with neurofibromatosis type 1. Eur J Nucl Med Mol Imaging.

[27] Tsai LL, Drubach L, Fahey F, Irons M, Voss S, Ullrich NJ (2012). [18F]-Fluorodeoxyglucose positron emission tomography in children with neurofibromatosis type 1 and plexiform neurofibromas: correlation with malignant transformation. J Neurooncol.

[28] Friedrich RE, Hartmann M, Mautner VF (2007). Malignant peripheral nerve Sheath tumors (MPNST) in NF1-affected children. Anticancer Res.

[29] Tahari AK, Chien D, Azadi JR, Wahl RL (2014). Optimum lean body formulation for correction of standardized uptake value in PET imaging. J Nucl Med.

[30] Kinahan PE, Fletcher JW (2010). Positron emission tomography-computed tomography standardized uptake values in clinical practice and assessing response to therapy. Semin Ultrasound, CT MRI.

[31] Broski SM, Johnson GB, Howe BM (2016). Evaluation of 18F-FDG PET and MRI in differentiating benign and malignant peripheral nerve sheath tumors. Skeletal Radiol.

[32] Bensaid B, Giammarile F, Mognetti T (2007). Intérêt de la tomographie par émission de positons au fluorodéoxyglucose 18 dans la détection des neurofibrosarcomes au cours de la neurofibromatose de type 1. Ann Dermatol Venereol.

[33] Benz MR, Czernin J, Dry SM (2010). Quantitative F18-fluorodeoxyglucose positron emission tomography accurately characterizes peripheral nerve sheath tumors as malignant or benign. Cancer.

[34] Cook GJR, Lovat E, Siddique M, Goh V, Ferner R, Warbey VS (2017). Characterisation of malignant peripheral nerve sheath tumours in neurofibromatosis-1 using heterogeneity analysis of (18)F-FDG PET. Eur J Nucl Med Mol Imaging.

[35] Lerman L, Zehou O, Ortonne N (2019). Interest of 18F-FDG PET/CT in neurofibromatosis type 1, 10-year experience from the national reference centre Henri-Mondor. Med Nucl.

[36] Nose H, Otsuka H, Otomi Y (2013). Correlations between F-18 FDG PET/CT and pathological findings in soft tissue lesions. J Med Invest.

[37] Reinert CP, Schuhmann MU, Bender B (2019). Comprehensive anatomical and functional imaging in patients with type I neurofibromatosis using simultaneous FDG-PET/MRI. Eur J Nucl Med Mol Imaging.

[38] Salamon J, Derlin T, Bannas P (2013). Evaluation of intratumoural heterogeneity on (1)(8)F-FDG PET/CT for characterization of peripheral nerve sheath tumours in neurofibromatosis type 1. Eur J Nucl Med Mol Imaging.

[39] Schwabe M, Spiridonov S, Yanik EL (2019). How effective are noninvasive tests for diagnosing malignant peripheral nerve sheath tumors in patients with neurofibromatosis type 1? Diagnosing MPNST in NF1 patients. Sarcoma.

[40] Warbey VS, Ferner RE, Dunn JT, Calonje E, O’Doherty MJ (2009). [18F]FDG PET/CT in the diagnosis of malignant peripheral nerve sheath tumours in neurofibromatosis type-1. Eur J Nucl Med Mol Imaging.

[41] Combemale P, Valeyrie-Allanore L, Giammarile F (2014). Utility of 18F-FDG PET with a semi-quantitative index in the detection of sarcomatous transformation in patients with neurofibromatosis type 1. PLoS ONE.

[42] Chhabra A, Soldatos T, Durand DJ, Carrino JA, McCarthy EF, Belzberg AJ (2011). The role of magnetic resonance imaging in the diagnostic evaluation of malignant peripheral nerve sheath tumors. Indian J Cancer.

[43] Karabatsou K, Kiehl T-R, Wilson DM, Hendler A, Guha A (2009). Potential role of 18fluorodeoxyglucose-positron emission tomography/computed tomography in differentiating benign neurofibroma from malignant peripheral nerve sheath tumor associated with neurofibromatosis 1. Neurosurgery.

[44] Ferner RE, Lucas JD, O’Doherty MJ (2000). Evaluation of 18fluorodeoxyglucose positron emission tomography (18FDG PET) in the detection of malignant peripheral nerve sheath tumours arising from within plexiform neurofibromas in neurofibromatosis 1. J Neurol Neurosurg Psychiatr.

[45] Chirindel A, Chaudhry M, Blakeley JO, Wahl R (2015). 18F-FDG PET/CT qualitative and quantitative evaluation in neurofibromatosis type 1 patients for detection of malignant transformation: comparison of early to delayed imaging with and without liver activity normalization. J Nucl Med.

[46] Bredella MA, Torriani M, Hornicek F (2007). Value of PET in the assessment of patients with neurofibromatosis type 1. AJR.

[47] Ducatman BS, Scheithauer BW, Piepgras DG, Reiman HM, Ilstrup DM (1986). Malignant peripheral nerve sheath tumors. A clinicopathologic study of 120 cases. Cancer J.

[48] Wong WW, Hirose T, Scheithauer BW, Schild SE, Gunderson LL (1998). Malignant peripheral nerve sheath tumor: analysis of treatment outcome. Int J Radiat Oncol Biol Phys.

