# Supplementary material for: Diagnostic value of 18F-FDG PET-CT in detecting malignant peripheral nerve sheath tumors among adult and pediatric neurofibromatosis type 1 patients
Source: J Neurooncol. 2022 Jan 13;156(3):559–67. doi: 10.1007/s11060-021-03936-y (PMC8860956; doi:10.1007/s11060-021-03936-y)
Supplement: Supplementary file 1 — Supplementary file1 (DOCX 68 kb) [file 11060_2021_3936_MOESM1_ESM.docx]

**Supplementary figure 1** Boxplots displaying differences in standard uptake value and tumor-to-liver ratio of semi-quantitative imaging markers for subgroups of MPNSTs and BPNSTs


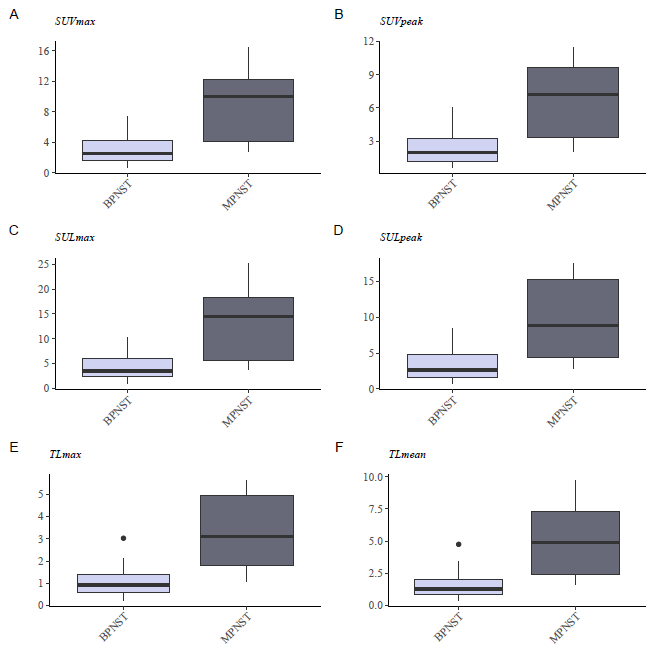


Boxplots displaying values of semi-quantitative imaging markers for subgroups MPNST and BPNST. A) SUVmax. B) SUVpeak. C) SULmax. D) SULpeak. E) TLmax. F) TLmean. Abbreviations: BPNST = benign peripheral nerve sheath tumor; MPNST = malignant peripheral nerve sheath tumor; SUV = standard uptake value; SUL = standard uptake value adjusted for lean body mass; TL = tumor-to-liver ratio.

**Supplementary figure 2** Boxplots displaying differences in delayed standard uptake value of semi-quantitative imaging markers for subgroups of MPNSTs and BPNSTs


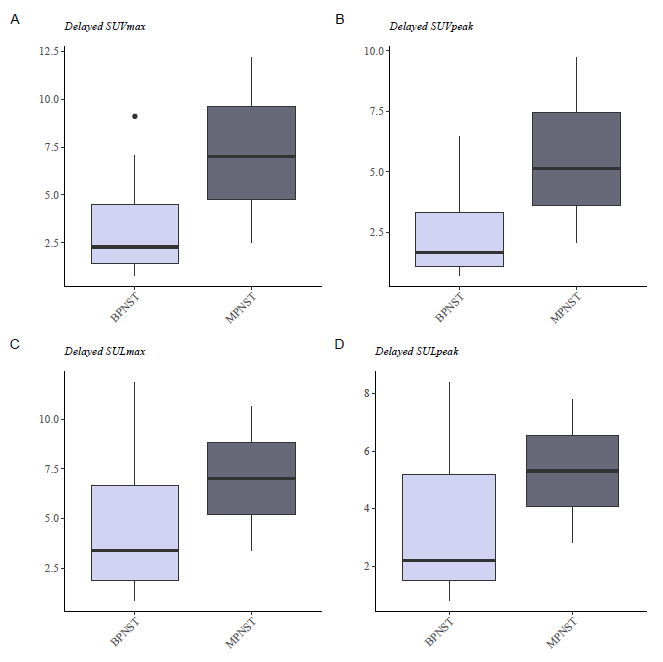


Boxplots displaying delayed values of semi-quantitative imaging markers for subgroups MPNST and BPNST. A) Delayed SUVmax. B) Delayed SUVpeak. C) Delayed SULmax. D) Delayed SULpeak. Abbreviations: BPNST = benign peripheral nerve sheath tumor; MPNST = malignant peripheral nerve sheath tumor; SUV = standard uptake value; SUL = standard uptake value adjusted for lean body mass.
