# Supplementary material for: Diagnostic value of 18F-FDG PET-CT in detecting malignant peripheral nerve sheath tumors among adult and pediatric neurofibromatosis type 1 patients
Source: J Neurooncol. 2022 Jan 13;156(3):559–67. doi: 10.1007/s11060-021-03936-y (PMC8860956; doi:10.1007/s11060-021-03936-y)
Supplement: Supplementary file 2 — Supplementary file2 (DOCX 25 kb) [file 11060_2021_3936_MOESM2_ESM.docx]

**Supplementary table 1** Threshold values of semi-quantitative imaging markers with optimal AUC, sensitivity, and specificity

|  |  | AUCmax | Sens 100% | Spec 100% |
| --- | --- | --- | --- | --- |
| *Overall* | **SUVmax** | 5.8 | 2.8 | 7.3 |
|  | **SUVpeak** | 5.0 | 2.0 | 6.1 |
|  | **SULmax** | 3.4 | 3.7 | 10.4 |
|  | **SULpeak** | 2.8 | 2.8 | 8.5 |
|  | **TLmax** | 1.7 | 1.0 | 2.1 |
|  | **TLmean** | 2.3 | 1.6 | 4.8 |
| *Adults* | **SUVmax** | 5.8 | 3.6 | 7.4 |
|  | **SUVpeak** | 5.0 | 2.8 | 6.1 |
|  | **SULmax** | 10.6 | 5.6 | 10.4 |
|  | **SULpeak** | 7.8 | 4.4 | 8.5 |
|  | **TLmax** | 3.1 | 1.0 | 2.2 |
|  | **TLmean** | 4.4 | 1.6 | 3.5 |
| *Children* | **SUVmax** | 2.8 | 2.8 | 6.5 |
|  | **SUVpeak** | 2.1 | 2.0 | 4.8 |
|  | **SULmax** | 3.4 | 3.7 | 8.9 |
|  | **SULpeak** | 2.8 | 2.8 | 6.6 |
|  | **TLmax** | 1.7 | 1.6 | 3.1 |
|  | **TLmean** | 2.3 | 2.3 | 4.8 |

Table showing threshold values of semi-quantitative imaging markers achieving highest AUC, sensitivity, and specificity. Abbreviations: AUC = area under the receiver operating curve; sens = sensitivity; spec = specificity; SUV = standard uptake value; SUL = standard uptake value adjusted for lean body mass; TLmax = tumor-to-liver maximal ratio; TLmean = tumor-to-liver mean ratio.

**Supplementary table 2** Semi-quantitative imaging markers threshold values offering 100% sensitivity and 100% specificity

|  |  | TP | FP | | FN | | | TN | | Sens | Spec | | PPV | | NPV | | | pLR | | | nLR |
| --- | --- | --- | --- | --- | --- | --- | --- | --- | --- | --- | --- | --- | --- | --- | --- | --- | --- | --- | --- | --- | --- |
| **SUVmax** | *Overall* | | | | | | | | | | | | | | | | | | | | |
|  | >2.8 | 10 | 27 | | 0 | | | 32 | | 1.00 | 0.54 | | 0.27 | | 1.00 | | | 2.19 | | | 0.00 |
|  | >3.0 | 9 | 26 | | 1 | | | 33 | | 0.90 | 0.56 | | 0.26 | | 0.97 | | | 2.05 | | | 0.18 |
|  | >3.5 | 8 | 22 | | 2 | | | 37 | | 0.80 | 0.63 | | 0.27 | | 0.95 | | | 2.16 | | | 0.32 |
|  | >4.0 | 7 | 16 | | 3 | | | 43 | | 0.70 | 0.73 | | 0.30 | | 0.93 | | | 2.59 | | | 0.41 |
|  | >4.5 | 7 | 11 | | 3 | | | 48 | | 0.70 | 0.81 | | 0.39 | | 0.94 | | | 3.68 | | | 0.37 |
|  | >5.0 | 7 | 7 | | 3 | | | 52 | | 0.70 | 0.88 | | 0.50 | | 0.95 | | | 5.83 | | | 0.34 |
|  | >5.5 | 7 | 6 | | 3 | | | 53 | | 0.70 | 0.90 | | 0.54 | | 0.95 | | | 7.00 | | | 0.33 |
|  | >6.0 | 6 | 4 | | 4 | | | 55 | | 0.60 | 0.93 | | 0.60 | | 0.93 | | | 8.57 | | | 0.43 |
|  | >7.3 | 6 | 0 | | 4 | | | 59 | | 0.60 | 1.00 | | 1.00 | | 0.94 | | |  | | | 0.40 |
|  | *Adults* | | | | | | | | | | | | | | | | | | | | |
|  | >3.6 | 8 | 17 | | 0 | | | 29 | | 1.00 | 0.63 | | 0.32 | | 1.00 | | | 2.71 | | | 0.00 |
|  | >7.4 | 6 | 0 | | 2 | | | 46 | | 0.75 | 1.00 | | 1.00 | | 0.96 | | |  | | | 0.25 |
|  | *Children* | | | | | | | | | | | | | | | | | | | | |
|  | >2.8 | 2 | 4 | | 0 | | | 9 | | 1.00 | 0.69 | | 0.33 | | 1.00 | | | 3.25 | | | 0.00 |
|  | >6.5 | 0 | 0 | | 2 | | | 13 | | 0 | 1.00 | |  | | 0.87 | | |  | | | 1.00 |
| **SUVpeak** | *Overall* | | | | | | | | | | | | | | | | | | | | |
|  | >2.0 | 10 | 28 | | | | 0 | 31 | | 1.00 | 0.53 | | 0.26 | | 1.00 | | | 2.11 | | | 0.00 |
|  | >6.1 | 6 | 0 | | | | 4 | 59 | | 0.60 | 1.00 | | 1.00 | | 0.94 | | |  | | | 0.40 |
|  | *Adults* | | | | | | | | | | | | | | | | | | | | |
|  | >2.8 | 8 | 18 | | | | 0 | 28 | | 1.00 | 0.61 | | 0.31 | | 1.00 | | | 2.56 | | | 0.00 |
|  | >6.1 | 6 | 0 | | | | 2 | 46 | | 0.75 | 1.00 | | 1.00 | | 0.96 | | |  | | | 0.25 |
|  | *Children* | | | | | | | | | | | | | | | | | | | | |
|  | >2.0 | 2 | 4 | | | | 0 | 9 | | 1.00 | 0.69 | | 0.33 | | 1.00 | | | 3.25 | | | 0.00 |
|  | >4.8 | 0 | 0 | | | | 2 | 13 | | 0 | 1.00 | |  | | 0.87 | | |  | | | 1.00 |
| **TLmax** | *Overall* | | | | | | | | | | | | | | | | | | | | |
|  | >1.0 | 10 | | 25 | | 0 | | | 33 | 1.00 | 0.57 | | 0.29 | | 1.00 | | | 2.32 | | | 0.00 |
|  | >2.1 | 5 | | 0 | | 5 | | | 58 | 0.50 | 1.00 | | 1.00 | | 0.92 | | |  | | | 0.50 |
|  | *Adults* | | | | | | | | | | | | | | | | | | | | |
|  | >1.0 | 8 | | 20 | | 0 | | | 25 | 1.00 | 0.56 | | 0.29 | | 1.00 | | 2.25 | | | 0.00 | |
|  | >2.2 | 6 | | 0 | | 2 | | | 45 | 0.75 | 1.00 | | 1.00 | | 0.96 | |  | | | 0.25 | |
|  | *Children* | | | | | | | | | | | | | | | | | | | | |
|  | >1.6 | 2 | | 2 | | 0 | | | 11 | 1.00 | 0.85 | 0.50 | | 0.10 | | 6.50 | | | 0.00 | | |
|  | >3.1 | 0 | | 0 | | 2 | | | 13 | 0 | 1.00 |  | | 0.87 | |  | | | 1.00 | | |
| **TLmean** | *Overall* | | | | | | | | | | | | | | | | | | | | |
|  | >1.6 | 10 | | 22 | | 0 | | | 36 | 1.00 | 0.62 | 0.31 | | 1.00 | | 2.64 | | | 0.00 | | |
|  | >2.0 | 9 | | 15 | | 1 | | | 43 | 0.90 | 0.74 | 0.38 | | 0.98 | | 3.46 | | | 0.14 | | |
|  | >2.5 | 7 | | 10 | | 3 | | | 48 | 0.70 | 0.83 | 0.41 | | 0.94 | | 4.12 | | | 0.36 | | |
|  | >3.0 | 6 | | 2 | | 4 | | | 56 | 0.60 | 0.97 | 0.75 | | 0.93 | | 20.00 | | | 0.41 | | |
|  | >4.8 | 5 | | 0 | | 5 | | | 58 | 0.50 | 1.00 | 1.00 | | 0.92 | |  | | | 0.50 | | |
|  | *Adults* | | | | | | | | | | | | | | | | | | | | |
|  | >1.6 | 8 | | 19 | | 0 | | | 26 | 1.00 | 0.58 | 0.30 | | 1.00 | | 2.37 | | | 0.00 | | |
|  | >3.5 | 6 | | 0 | | 2 | | | 45 | 0.75 | 1.00 | 1.00 | | 0.96 | |  | | | 0.25 | | |
|  | *Children* | | | | | | | | | | | | | | | | | | | | |
|  | >2.3 | 2 | | 2 | | 0 | | | 11 | 1.00 | 0.85 | 0.50 | | 0.10 | | 6.50 | | | 0.00 | | |
|  | >4.8 | 0 | | 0 | | 2 | | | 13 | 0 | 1.00 |  | | 0.87 | |  | | | 1.00 | | |
| **SULmax** | *Overall* | | | | | | | | | | | | | | | | | | | | |
|  | >3.7 | 9 | | 29 | | 0 | | | 30 | 1.00 | 0.51 | 0.24 | | 1.00 | | 2.03 | | | 0.00 | | |
|  | >10.4 | 5 | | 0 | | 4 | | | 59 | 0.56 | 1.00 | 1.00 | | 0.94 | |  | | | 0.44 | | |
|  | *Adults* | | | | | | | | | | | | | | | | | | | | |
|  | >5.6 | 7 | | 18 | | 0 | | | 28 | 1.00 | 0.61 | 0.28 | | 1.00 | | 2.56 | | | 0.00 | | |
|  | >10.4 | 5 | | 0 | | 2 | | | 46 | 0.71 | 1.00 | 1.00 | | 0.96 | |  | | | 0.29 | | |
|  | *Children* | | | | | | | | | | | | | | | | | | | | |
|  | >3.7 | 2 | | 5 | | 0 | | | 8 | 1.00 | 0.62 | 0.29 | | 1.00 | | 2.60 | | | 0.00 | | |
|  | >8.9 | 0 | | 0 | | 2 | | | 13 | 0 | 1.00 |  | | 0.87 | |  | | | 1.00 | | |
| **SULpeak** | *Overall* | | | | | | | | | | | | | | | | | | | | |
|  | >2.8 | 9 | | 28 | | 0 | | | 31 | 1.00 | 0.53 | 0.24 | | 1.00 | | 2.11 | | | 0.00 | | |
|  | >8.5 | 5 | | 0 | | 4 | | | 59 | 0.56 | 1.00 | 1.00 | | 0.94 | |  | | | 0.44 | | |
|  | *Adults* | | | | | | | | | | | | | | | | | | | | |
|  | >4.4 | 7 | | 17 | | 0 | | | 29 | 1.00 | 0.63 | 0.29 | | 1.00 | | 2.71 | | | 0.00 | | |
|  | >8.5 | 5 | | 0 | | 2 | | | 46 | 0.71 | 1.00 | 1.00 | | 0.96 | |  | | | 0.29 | | |
|  | *Children* | | | | | | | | | | | | | | | | | | | | |
|  | >2.8 | 2 | | 4 | | 0 | | | 9 | 1.00 | 0.69 | 0.33 | | 1.00 | | 3.25 | | | 0.00 | | |
|  | >6.6 | 0 | | 0 | | 2 | | | 13 | 0 | 1.00 |  | | 0.87 | |  | | | 1.00 | | |

Table showing threshold values of semi-quantitative imaging markers with 100% sensitivity and 100% specificity. Abbreviations: FN = false negatives; FP = false positives; nLR = negative likelihood ratio; NPV = negative predictive value; pLR = positive likelihood ratio; PPV = positive predictive value; sens = sensitivity; spec = specificity; SUL = standard uptake value adjusted for lean body mass; SUV = standard uptake value; TLmax = tumor-to-liver maximal ratio; TLmean = tumor-to-liver mean ratio; TN = true negatives; TP = true positives.

**Supplementary table 3** Threshold values combining TLmean and SUVmax

|  |  | TP | FP | FN | TN | Sens | Spec | PPV | NPV | pLR | nLR |
| --- | --- | --- | --- | --- | --- | --- | --- | --- | --- | --- | --- |
|  | **Combined AND** | | | | | | | | | | |
|  | TLmean > 2.0 + SUVmax > 3.5 | 7 | 8 | 3 | 50 | 0.70 | 0.86 | 0.47 | 0.94 | 5.00 | 0.35 |
|  | TLmean > 2.3 + SUVmax > 5.0 | 6 | 1 | 4 | 57 | 0.60 | 0.98 | 0.86 | 0.93 | 30.00 | 0.41 |
|  | TLmean > 2.0 + SUVmax > 5.5 | 6 | 1 | 4 | 58 | 0.60 | 0.98 | 0.86 | 0.94 | 30.00 | 0.41 |
|  | TLmean > 2.0 + SUVmax > 6.0 | 6 | 1 | 4 | 58 | 0.60 | 0.98 | 0.86 | 0.94 | 30.00 | 0.41 |
|  | TLmean > 1.5 + SUVmax > 5.5 | 6 | 1 | 4 | 58 | 0.60 | 0.98 | 0.86 | 0.94 | 30.00 | 0.41 |
|  | TLmean > 1.5 + SUVmax > 6.0 | 6 | 1 | 4 | 58 | 0.60 | 0.98 | 0.86 | 0.94 | 30.00 | 0.41 |
|  | **Combined OR** | | | | | | | | | | |
|  | Subset Tlmean >2.0 / TLmean <2.0 & SUVmax >3.5 | 10 | 22 | 0 | 36 | 1.00 | 0.62 | 0.31 | 1.00 | 2.64 | 0.00 |
|  | Subset Tlmean >2.3 / TLmean <2.3 & SUVmax >3.5 | 10 | 21 | 0 | 37 | 1.00 | 0.64 | 0.32 | 1.00 | 2.76 | 0.00 |
|  | Subset Tlmean >2.0 / TLmean <2.0 & SULmax >5.5 | 10 | 22 | 0 | 36 | 1.00 | 0.62 | 0.31 | 1.00 | 2.64 | 0.00 |
|  | Subset Tlmean >4.0 / TLmean <4.0 & SULmax >3.5 | 10 | 30 | 0 | 28 | 1.00 | 0.48 | 0.25 | 1.00 | 1.93 | 0.00 |

Table showing threshold values combining SUVmax and TLmean. Abbreviations: FN = false negatives; FP = false positives; nLR = negative likelihood ratio; NPV = negative predictive value; pLR = positive likelihood ratio; PPV = positive predictive value; sens = sensitivity; spec = specificity; SUL = standard uptake value adjusted for lean body mass; SUV = standard uptake value; TLmean = tumor-to-liver mean ratio; TN = true negatives; TP = true positives.
